# Internal fixation vs manual reduction in the treatment of ankle fracture healing and inflammation: A case–control study

**DOI:** 10.1097/MD.0000000000041071

**Published:** 2025-01-10

**Authors:** Yaheng Wei, Zuoming Yang

**Affiliations:** a Hebei Province Tangshan Second Hospital Trauma Five, Hebei, China.

## Abstract

Ankle fractures are among the most common bone injuries, which are often accompanied by soft tissue injuries. Proper management of these fractures is crucial to promote healing and minimize complications. This study explores the effects of 2 treatment methods for ankle fractures: open reduction and internal fixation and manual reduction followed by plaster external fixation. A retrospective analysis was conducted on 124 patients with ankle fractures admitted between March 2020 and September 2022. Patients were divided into 2 groups: 62 received internal fixation and 62 received manual reduction with plaster external fixation. The study evaluated various clinical outcomes, including treatment effectiveness, recovery times, the incidence of nonunion, ankle joint function and inflammatory factors, and complication rates. The internal fixation group showed a significantly higher effective treatment rate (96.77%) compared to the non-internal fixation group (85.48%). After treatment, the internal fixation group had significantly lower medial malleolus space and talus tilt angles, indicating better fracture alignment. The internal fixation group also had shorter treatment, postoperative recovery, and functional recovery times. Furthermore, the incidence of nonunion and complications was lower in the internal fixation group. Inflammatory markers such as interleukin-6 (IL-6), C-reactive protein (CRP), and interleukin-8 (IL-8) decreased significantly in the internal fixation group compared to the non-internal fixation group. Internal fixation is more effective than manual reduction and plaster external fixation for treating ankle fractures. It leads to better fracture healing, shorter recovery times, and fewer complications, including nonunion. Manual reduction with external fixation remains a viable option but may be associated with a higher risk of nonunion and delayed healing. Early and effective management of soft tissue injuries is crucial for improving treatment outcomes in ankle fractures.

## 1. Introduction

Ankle fracture is the most usual type of bone lesion. The annual global incidence of ankle fracture is about 70 to 180 per 100,000 people,^[1,2]^ particularly when combined with fragility fractures, are more common in individuals over the age of 50, contributing significantly to both social and economic burdens.^[3]^ The ankle joint is 1 of the main weight-bearing joints of the human body.^[4]^ Therefore, it is crucial to choose appropriate treatment methods to promote fracture healing early. Ankle fractures are often accompanied by severe soft tissue damage. Correct handling of soft tissue injuries is a basic element in the therapy of ankle fractures.^[5]^ If the soft tissue injuries may cause ankle pain, osteochondral damage, and serious complications, and even aggravate the patient’s condition and affect the patient’s quality of life.^[6]^ For example, when manual reduction is performed, local qi and blood should be dredged by acupoints, local muscle spasm should be relieved, and ligaments and surrounding supporting bands should be loosened. During surgical treatment, attention should be paid to carefully separating and protecting nerves, and a slightly curved incision can be made, and the incision should not be too deep to avoid aggravating soft tissue injury.^[7–10]^ Therefore, patients should be given certain reduction and fixation treatments to decrease the risk of skin damage caused by dislocation fractures and provide good functional recovery. Manual reduction and surgical reduction are the main clinical treatments for ankle fractures.^[11,12]^ However, there is still some disagreement about which treatment method to choose, and both of them can play a certain role. However, after manual reduction treatment, it may be displaced again, and the external fixation after manual reduction is not firm, which may lead to abnormal healing or even non-healing, affecting the treatment effect. Surgical treatment is more important for the choice of operation opportunity. Once the limb at the fracture is obviously swollen, localized and even adhered, it will make anatomical alignment difficult to achieve and may have great sequelae. Based on this, sufferers with ankle fractures diagnosed and admitted to our hospital were regarded as the study subjects in this study. The sufferers were treated with plaster external fixation after traditional manual reduction and open reduction and internal fixation respectively, aiming to explore its effect on the clinical treatment indicators and secondary treatment of patients. The impact of treatment on the incidence of nonunion, excellent and good ankle joint function, inflammatory factor indicators and incidence of complications, to give a better reference for the therapy of clinical illnesses.

## 2. Methods

### 2.1. Basic information

This research was approved by the hospital ethics committee (approval no.: PZ-HNSZYY-2022-029). The medical records of 124 sufferers with ankle fractures were selected for retrospective analysis, and their admission time ranged from March 2020 to September 2022. All research subjects who met the inclusion criteria were divided into 2 groups based according to the treatment methods, 62 instances in the non-internal fixation 1 and 62 instances in the internal fixation 1. It had no statistically obvious distinction in basic information such as age, fracture site, gender, pain degree, swelling degree, and cause of injury between the 2 groups of sufferers (*P > *.05) (see Table 1 for more information).

**Table 1:** Basic data analysis of the 2 groups [n (%), ($\bar{x}\pm s$)].

Group	n	Age (yr)	Fracture site (%)		Gender (%)	
			Left side	Right side	Male	Female
Internal fixation group	62	37.95 ± 3.93	25 (40.32)	37 (59.68)	40 (64.52)	22 (35.48)
Non-internal fixation group	62	36.53 ± 4.12	23 (37.10)	39 (62.90)	42 (67.74)	20 (32.26)
*χ^2^/t*		1.958	0.136		0.144	
*P*		.052	.712		.704	

Group	n	Pain level (min)	Degree of swelling (min)	Cause of injury (%)		
				Walking impairment	Traffic accident	Other
Internal fixation group	62	2.71 ± 0.28	7.51 ± 0.95	29 (46.77)	16 (25.81)	17 (27.42)
Non-internal fixation group	62	2.67 ± 0.29	7.35 ± 0.74	31 (50.00)	11 (17.74)	20 (32.26)
*χ ^2^/t*		0.748	1.069	1.236		
*P*		.456	.287	.539		

### 2.2. Inclusion criteria

Sufferers have ankle fracture confirmed by imaging; patients are between 20 and 65 years old; patients have clear cognitive function and can cooperate with relevant work in the research; the patient and his family members have a clear understanding of the relevant content of the study and sign the consent form for confirmation; the patient has a significant history of trauma.

### 2.3. Exclusion criteria

Patients with open fractures were excluded due to the increased risk of infection and the need for different treatment approaches compared to closed fractures. Patients with severe systemic conditions, such as heart disease, diabetes, or immune disorders. Patients with osteoporosis or other metabolic bone disorders were excluded due to the potential for impaired bone healing and the challenges associated with standard treatment methods. Patients under 18 years old were excluded. Patients with preexisting severe ankle joint conditions, such as arthritis or congenital deformities. Pregnant or breastfeeding women were excluded due to potential risks associated with certain treatments and the physiological changes during pregnancy that may influence healing. Patients with significant associated soft tissue injuries, such as vascular, nerve, or tendon damage. Patients with known allergies to the materials used in the treatment, such as metal implants (e.g., titanium alloys) or medications (e.g., anesthetics), were excluded to prevent adverse reactions and ensure patient safety. Patients with cognitive impairments or psychiatric disorders that hinder adherence to treatment protocols or follow-up.

### 2.4. Treatment

Internal fixation group: open reduction and internal fixation was performed, with the patient in a supine position and combined spinal-epidural anesthesia. The incision method was selected according to the sufferer’s fracture situation, and the fracture area within the field of view was cleaned to “posterior side.” Reduction and fixation are performed in the order of “lateral to medial”; cancellous bone screws are given to the posterior malleolus, hollow cancellous bone screws are given to the lateral malleolus, and absorbable screws are given to the medial malleolus. During the process, paying attention to avoiding damage to the ankle ligaments, large/small saphenous veins, and sural nerves, and suture the incision; Postoperative joint training and regular X-ray testing to observe sufferer healing. All surgical procedures in this study were performed by a single treatment team with extensive surgical experience. Non-internal fixation group: Traditional manual reduction followed by plaster external fixation was performed. Placing the sufferer in a supine position. After debridement and suturing of the fracture site, the patient’s knee joint was flexed at an angle of approximately 90°, on the outside of the affected limb. To pull the affected limb upward to resist traction; hold the front of the foot with 1 hand, support the heel with the other hand, and resist traction downward along the original deformity. Correcting the rotational displacement during the traction process, and fixing it below the knee joint with a plaster bandage. The fixation time is 4 to 8 weeks. After surgery, the patient’s reduction status will be judged based on the X-ray examination results, and functional exercises will be performed based on the patient’s recovery status.

### 2.5. Observation indicators

Clinical effect: Analyzing the clinical effect of treatment for patients with ankle fractures, including ineffectiveness, marked effectiveness and cure, and calculate the effective rate. Effective rate (%) = (significantly effective + cured) ÷ total number of cases (62 cases) × 100%. X-ray was used to detect the changes of 2 groups of influential indexes, including medial malleolus space and talus tilt angle (TT). Imaging tests will be conducted at 3 to 4 days, 4 weeks, 3 months, 6 months, 9 months, and 1-year post-surgery. Clinical treatment indicators: Recording and comparing the changes in clinical indicators of sufferers received in internal fixation and those treated without internal fixation, including treatment time, bleeding volume, postoperative recovery time and functional recovery time. Secondary treatment and the incidence of nonunion: Observing the incidence of secondary treatment and nonunion in patients within 6 months after surgery. Excellent and good ratio of ankle joint function: The excellent and good ratio of ankle joint function is evaluated using the American Orthopedic Foot and Ankle Society score,^[13]^ which includes 3 parts: function, pain, and affected foot force line. A point of < 60 is scored as poor, and a point of 60 to 75 is considered normal, a point of 76 to 90 were scored as good, and a point of ≥ 91 were scored as excellent. The excellent and good rate was calculated, and the excellent and good rate (%) = (good + excellent) total number of cases (62 cases) × 100%. Detection of inflammatory factor indicators: Enzyme-linked immunosorbent assay was used to detect changes in patients’ serum inflammatory factor indicators before treatment and 6 months after treatment, including interleukin-6 (IL-6), C-reactive protein (CRP), and interleukin-8 (IL-8). 4.5mL of median elbow venous blood was taken from patients with fasting for more than 8 hours, placed in an anticoagulant vacuum tube, left standing, centrifuged at a centrifugal speed of 3000r/min and a centrifugal force of 200 g for 15 minutes, and the supernatant was taken and stored in an ultra-low temperature refrigerator. Put the serum sample and the standard in the enzyme-labeled plate respectively, add the sealing membrane to react at 37°C, suck up the liquid in the plate, add the antibody of the detection index respectively, incubate at room temperature for 60 min, rinse with buffer, add the working liquid to incubate at room temperature for 30 min, rinse with buffer, add the chromogenic agent, incubate at room temperature in the dark for 20 min, add the stop liquid, and detect at 510 nm with an enzyme-labeled instrument. Complication analysis: Observing the occurrence of complications during or after treatment, and calculate the complication rate.

### 2.6. Statistical methods

All data analyzes in this study were analyzed using SPSS24.0. Count data such as clinical effects, secondary treatment and nonunion rates, excellent and good ankle function rates, and complication rates were expressed by (n [%]), pairwise comparisons were made through independent samples and the *χ^2^* test was used; clinical treatment indicators, inflammatory factors and other measurement data were expressed through ($\bar{x}\pm s$), and pairwise comparisons were made through independent samples *t* tests, and statistical results of *P* < .05 were considered distinctions had statistical obviousness.

## 3. Results

### 3.1. Analysis of the clinical effects of the 2 groups

**Figure 1.**
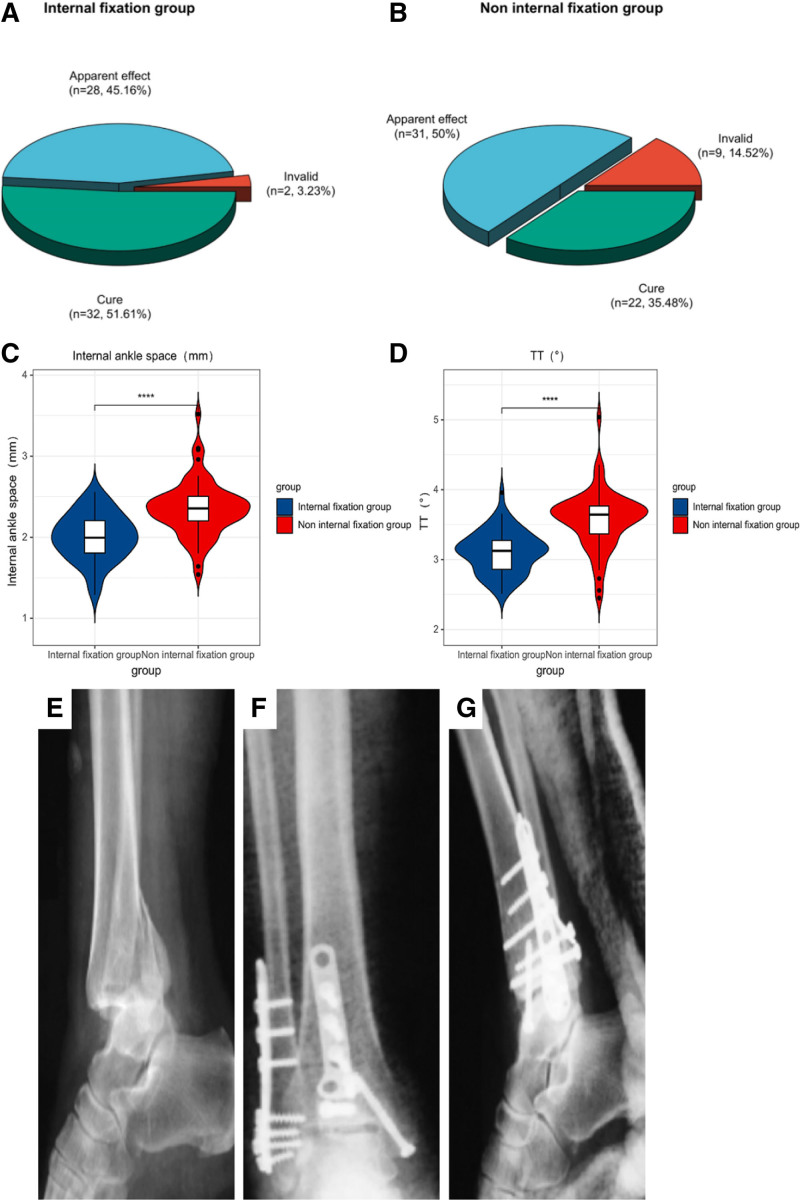
(A and B) Distribution of clinical effects between the 2 groups; (C and D) distribution of 2 sets of imaging indicators. (**** means *P* < .001 compare to the control 1 after intervention); (E–G) typical pictures of patients with ankle fracture. (E) preoperative image; (F and G) postoperative image.

The average length of hospital stay for patients in the surgical group was 4.5 ± 1.5 days. The effective ratio of treatment in the internal fixation 1 was 96.77% (marked efficiency 45.16% + cure rate 51.61%), which was significantly higher than the effective rate of 85.48% (marked efficiency 50.00% + cure rate 35.48%) in the non-internal fixation group, there is a clear distinction between the 2 groups (*P* < .05). There was no significant difference in medial malleolus space and TT level between the internal fixation group and the non-internal fixation group before treatment (*P* > .05). After treatment, the medial malleolus space and TT level of patients in the internal fixation group and the non-internal fixation group were significantly lower than those before treatment, and there were significant differences before and after treatment (*P* < .05). The above indexes in the internal fixation group were significantly lower than those in the non-internal fixation group, and there was a significant difference between the 2 groups (*P* < .05) (see Table 2 and Fig. 1).

**Table 2:** Analysis and comparison of clinical effects between the 2 groups [n (%)].

Group	n	Invalid	Effective	Cure	Efficient
Internal fixation group	62	2 (3.23)	28 (45.16)	32 (51.61)	60 (96.77)
Non-internal fixation group	62	9 (14.52)	31 (50.00)	22 (35.48)	53 (85.48)
*χ ^2^*					4.888
*P*					.027

Group	n	Internal ankle space (mm)		TT (°)	
		Before treatment	After treatment	Before treatment	After treatment
Internal fixation group	62	4.76 ± 1.59	2.00 ± 0.29	10.64 ± 3.53	3.11 ± 0.30
Non-internal fixation group	62	4.84 ± 1.50	2.35 ± 0.34	10.83 ± 3.74	3.57 ± 0.42

TT = talus tilt angle.

### 3.2. Analysis of clinical treatment indicators of the 2 groups

**Figure 2.**
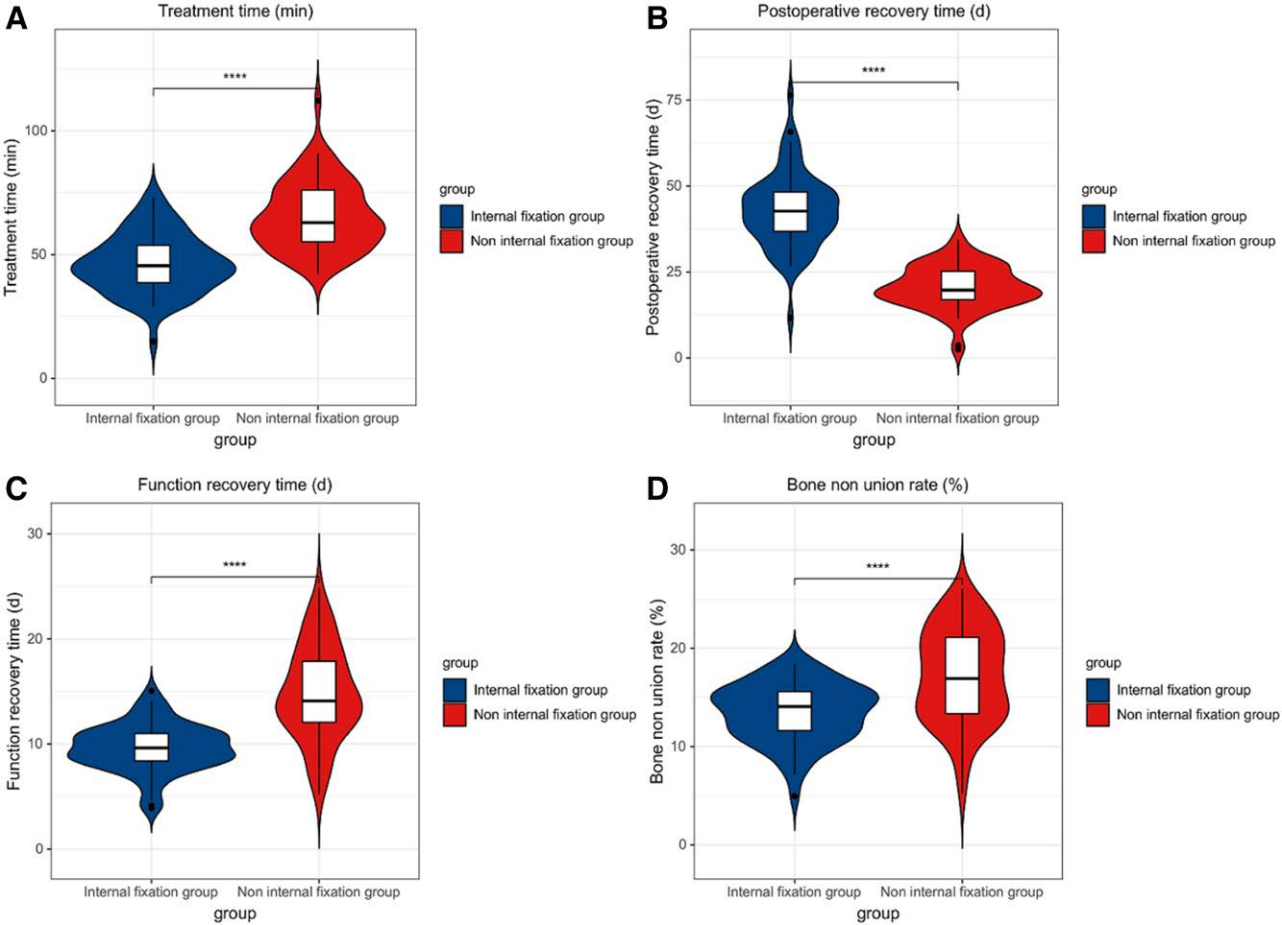
Distribution of clinical treatment indicators between the 2 groups (**** means *P* < .001 compare to the control 1 after intervention).

Compared with the non-internal fixation 1, the therapy time, postoperative recovery time and functional recovery time of sufferers in the internal fixation 1 were clearly shortened. It had a clear distinction between the 2 groups (*P* < .05). The amount of bleeding in the internal fixation 1 was clearly increased, and there was a significant difference between the 2 groups (*P* < .05) (see Table 3 and Fig. 2).

**Table 3:** Analysis and comparison of clinical treatment indicators between the 2 groups ($\bar{x}\pm s$).

Group	n	Treatment time (min)	Bleeding volume (mL)	Postoperative recovery time (d)	Functional recovery time (d)
Internal fixation group	62	46.30 ± 11.82	42.90 ± 10.81	9.66 ± 2.25	13.46 ± 2.94
Non-internal fixation group	62	65.73 ± 13.79	20.52 ± 6.25	14.82 ± 4.40	17.22 ± 4.73
*χ ^2^*		8.426	14.113	8.227	5.318
*P*		<.001	<.001	<.001	<.001

### 3.3. Analysis of the incidence of secondary treatment and nonunion in the 2 groups

**Figure 3.**
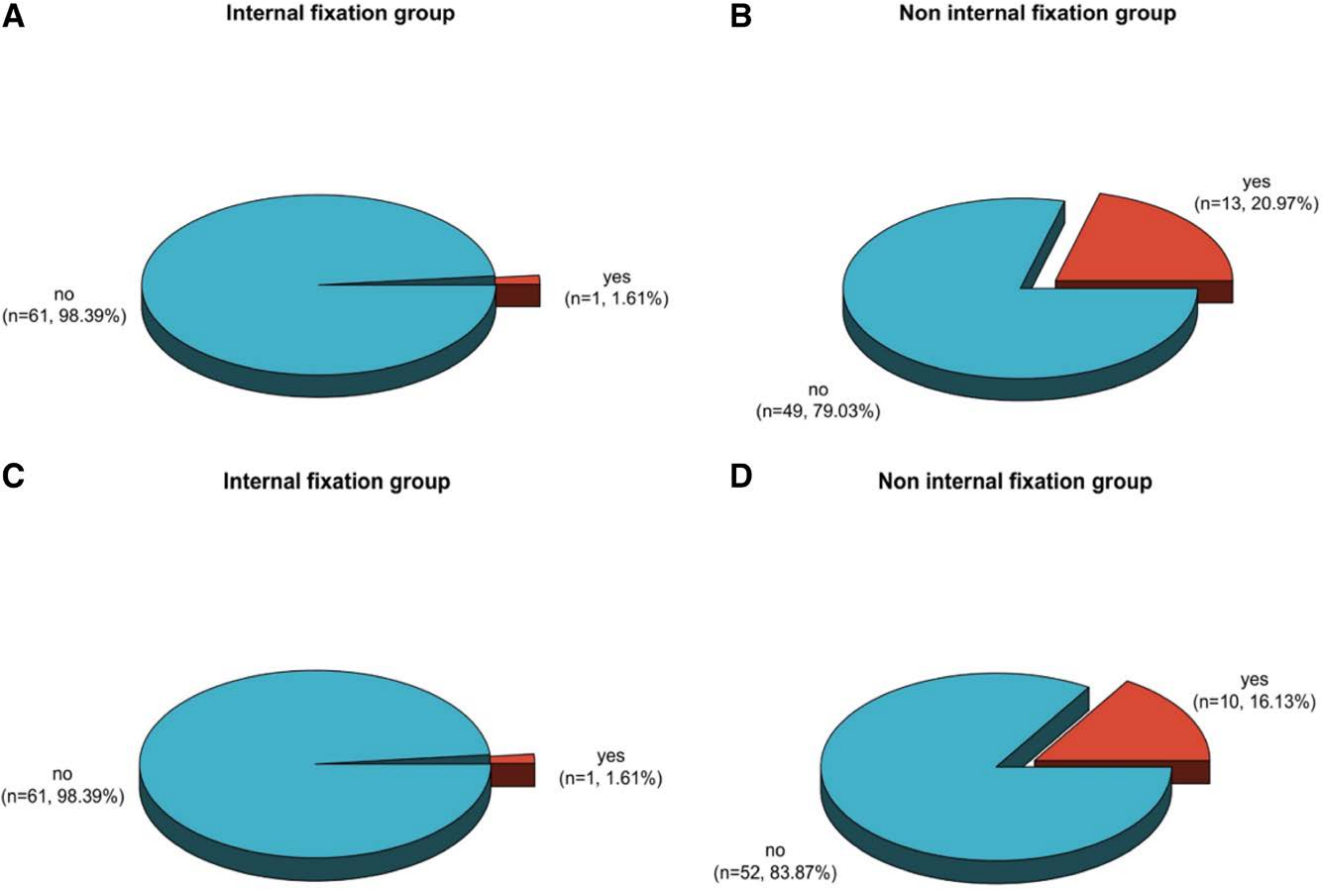
(A and B) Comparative distribution chart of the incidence of nonunion between the 2 groups; (C and D) Comparative distribution chart of secondary treatment incidence rates between the 2 groups.

In comparison of the frequency of nonunion (20.97%) and secondary therapy ratio (16.13%) in the non-internal fixation 1, the frequency of nonunion (1.61%) and secondary therapy ratio (1.61%) in the internal fixation 1 was both clearly decreased, and it had a clear distinction between the 2 groups (*P* < .05) (see Table 4 and Fig. 3).

**Table 4:** Analysis and comparison of the frequency of secondary therapy and nonunion among the 2 groups [n (%)].

Group	n	Nonunion rate	Second treatment rate
Internal fixation group	62	1 (1.61)	1 (1.61)
Non-internal fixation group	62	13 (20.97)	10 (16.13)
*χ ^2^*		11.595	8.081
*P*		.001	.004

### 3.4. Analysis of the excellent and good ratio of ankle joint function in the 2 groups

**Figure 4.**
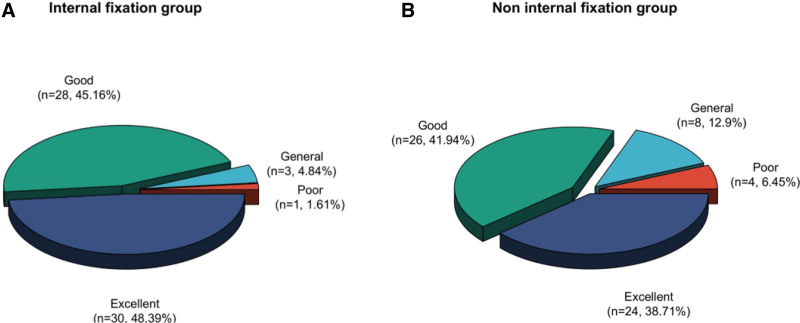
Distribution of excellent and good ankle function rates between the 2 groups.

In comparison of the excellent and good ratio of 80.65% (excellent ratio 38.71% + good ratio 41.94%) in the non-internal fixation 1, the excellent and good ratio of sufferers in the internal fixation 1 was 93.55% (excellent ratio 48.39% + good ratio 45.16%), which was both clearly increased, and it had a clear distinction between the 2 groups (*P* < .05) (see Table 5 and Fig. 4).

**Table 5:** Analysis and comparison of excellent and good ratios of ankle joint function between the 2 groups [n (%)].

Group	n	Poor	Generally	Good	Excellent	Excellent rate
Internal fixation group	62	1 (1.61)	3 (4.84)	28 (45.16)	30 (48.39)	58 (93.55)
Non-internal fixation group	62	4 (6.45)	8 (12.90)	26 (41.94)	24 (38.71)	50 (80.65)
χ^2^						4.593
*P*						.032

### 3.5. Analysis of inflammatory factor indicators between the 2 groups

**Figure 5.**
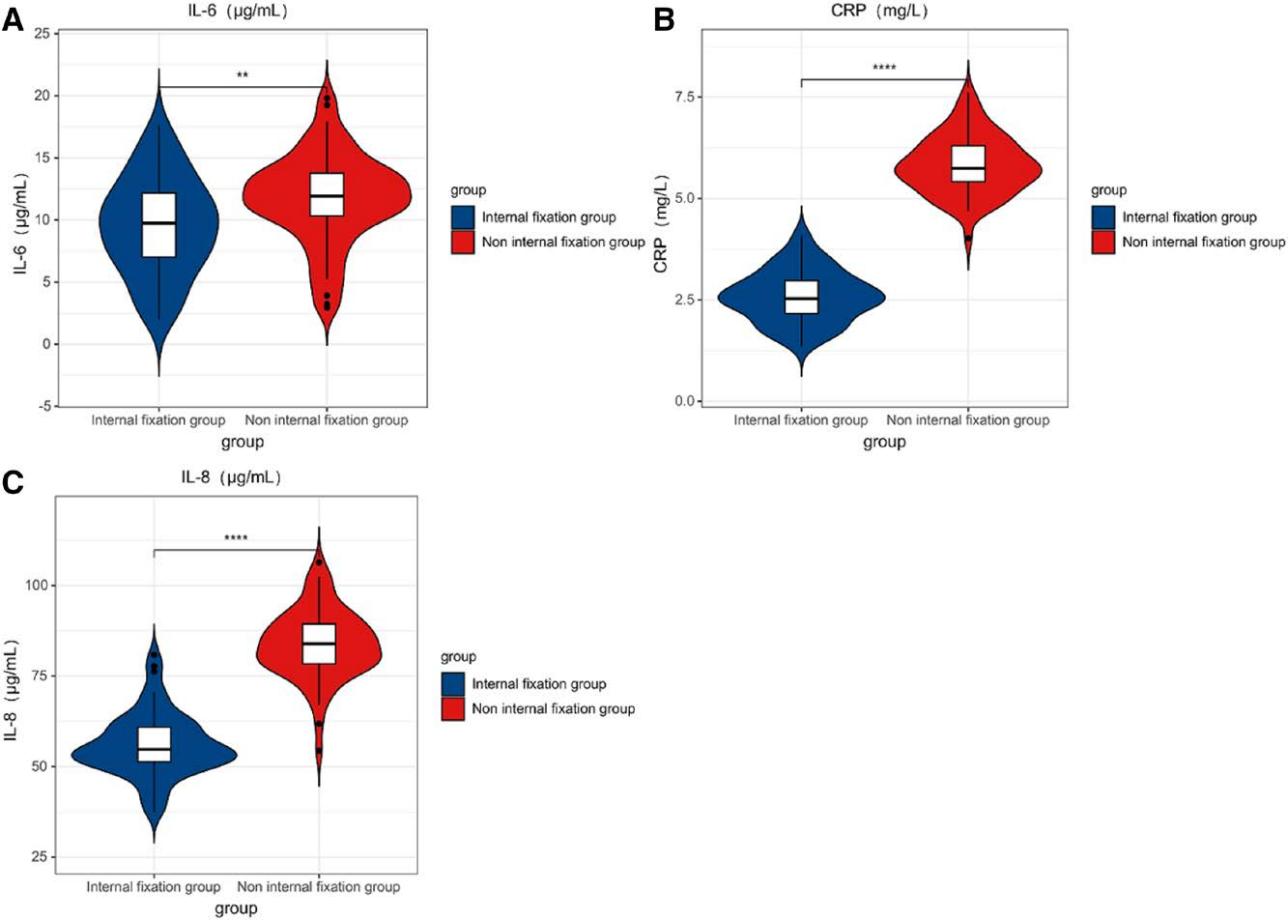
Distribution of inflammatory factor indicators in patients (** means *P* < .01 compare to the control 1 after intervention, **** means *P* < .001 compare to the control 1 after intervention).

It had no clear distinction in IL-6, CRP, and IL-8 levels between the internal fixation 1 and the non-internal fixation 1 at pre-therapy (*P* > .05); after therapy, it had no clear distinction between the internal fixation 1 and the non-internal fixation 1. The patient’s IL-6, CRP, and IL-8 levels were clearly less than those before therapy, and it had obvious distinctions before and after therapy (*P* < .05); and the above indicators in the internal fixation 1 were clearly less than the non-internal fixation 1, and it had clear distinctions between the 2 groups. (*P* < .05) (see Table 6 and Fig. 5).

**Table 6:** Analysis and comparison of inflammatory factor indicators between the 2 groups ($\bar{x}\pm s$).

Group	n	IL-6 (μg/mL)		CRP (mg/L)		IL-8 (μg/mL)	
		Before treatment	After treatment	Before treatment	After treatment	Before treatment	After treatment
Internal fixation group	62	60.29 ± 9.71	9.53 ± 3.91	11.83 ± 2.30	2.59 ± 0.63	117.74 ± 11.35	55.92 ± 8.73
Non-internal fixation group	62	60.97 ± 9.65	11.71 ± 3.62	11.24 ± 2.73	5.82 ± 0.72	120.25 ± 12.95	83.72 ± 9.60
*t*		0.349	3.235	1.300	26.593	1.147	16.871
*P*		.782	.002	.196	<.001	.253	<.001

CRP = C-reactive protein, IL-6 = interleukin-6, IL-8 = interleukin-8.

### 3.6. Analysis of complications of patient treatment

The complication ratio of sufferers in the internal fixation 1 was 4.84% (infection rate 1.61% + subcutaneous hematoma rate 3.23%) compared with the non-internal fixation 1 (infection frequency ratio 3.23%+ischemic necrosis frequency ratio 4.84%+healing deformity frequency ratio 4.84%+subcutaneous hematoma frequency ratio 8.06%) was clearly decreased, and it had a clear distinction between the 2 groups (*P* < .05) (see Table 7).

**Table 7:** Analysis and comparison of complications of patient treatment [n (%)].

Group	n	Infect	Avascular necrosis	Healing deformity	Subcutaneous hematoma	Total
Internal fixation group	62	1 (1.61)	0 (0.00)	0 (0.00)	2 (3.23)	3 (4.84)
Non-internal fixation group	62	2 (3.23)	3 (4.84)	3 (4.84)	5 (8.06)	13 (20.97)
χ^2^						7.176
*P*						.007

## 4. Discussion

Ankle fracture is 1 of the most usual and commonly treated injuries by clinical orthopedic surgeons. Patients often present with immediate ankle pain, local or general swelling, bruises, joint deformity, and inability to bear weight.^[14,15]^ Studies have shown that more than half of lower limb fractures are ankle fractures, and with the aging of the population, the frequency of ankle fractures has increased, increasing the economic burden of patients and affecting their lives.^[16]^ In recent years, the treatment of ankle joint is mainly external fixation, but the stability of external fixation is poor, secondary injuries often occur, and postoperative complications occur more.^[17]^ As a new type of fracture fixation method with less injuries, internal fixation has been gradually and widely used in the treatment of fractures, and achieved good results. In this study, the internal fixation method first repairs the bone structure damage to restore the normal anatomical length of the lateral malleolus, and corrects it by axial rotation, and uses cancellous bone nails and absorbable screws to fix it, which can improve the stability of the ankle joint while better fixing it. This study mainly discusses and analyzes the application of internal fixation in ankle fracture, so as to provide some theoretical reference for clinical treatment.

Maintaining fracture healing and restoring ankle function are the basic goals of clinical treatment of ankle fractures.^[18]^ Surgical intervention for ankle fractures usually has positive results.^[19]^ With the advancement of implants and surgical methods, the treatment effect of ankle fractures has been significantly improved, and more than 80% of patients have achieved good treatment effects.^[20]^ External fixation and internal fixation are the main treatments for ankle fractures. Among them, the principle of external fixation after traditional manual reduction is to take the opposite direction to the injury mechanism and promote the recovery of the bone fragments by manual pushing.^[21]^ Open reduction and internal fixation can achieve significant results in the treatment of ankle fractures. It could compensate for the shortcomings of biomechanical fixation from a biomechanical perspective, effectively enhance ankle stability, and accurately decrease the ankle joint, laying a solid foundation for the recovery of sufferers in the later stage.^[22–25]^ In this study, by analyzing the therapeutic effects and clinical treatment index changes of internal fixation and non-internal fixation on ankle fractures, it was found that the therapy effect of the internal fixation 1 was clearly better than the non-internal fixation 1, and the therapy time, postoperative recovery time, and functional recovery time were clearly shorter than the nonfixed 1. This shows that internal fixation treatment can have a positive function in promoting postoperative fracture healing and promoting early recovery of sufferers. In addition, the composite ligaments surrounding the ankle joint play a vital role in maintaining joint stability, and ligament reconstruction can be effectively accomplished through internal fixation. This is 1 of the key factors contributing to the success of internal fixation surgery.^[26]^ However, the amount of bleeding in the internal fixation 1 was greater than the non-internal fixation 1. The reason may be that open reduction places a larger bed on the patient, resulting in more bleeding. In addition, the outcomes of this research found that the internal fixation 1 had a lower incidence of secondary treatment and nonunion, and a higher rate of excellent ankle joint function. To explore the reasons, open reduction has a clearer field of vision and can better achieve the purpose of reduction. Compared with manual reduction, open reduction and internal fixation have higher stability and lower possibility of displacement again.^[27–29]^

Successful fracture healing is according to the carefully coordinated interaction between inflammatory cells and bone-forming cells. The inflammatory response is 1 of the important factors that aggravates the patient’s pain and delays the patient’s recovery.^[30–32]^ Inflammation is closely related to bone formation related to fracture healing and heterotopic ossification.^[33]^ Research has found that when a joint is damaged, an obvious inflammatory response occurs in the joint, and the expression of inflammatory cytokines increases significantly.^[34]^ The results of this study found that after therapy, the IL-6, CRP, and IL-8 levels in the internal fixation 1 were clearly less than the non-internal fixation 1. By analyzing the reasons, open reduction and internal fixation can protect the blood transmission of bones and periosteum to the greatest extent, promote fracture healing, shorten the fracture healing time on the basis of ensuring joint function, effectively reduce bacterial invasion, and thus inhibit the occurrence of inflammatory reaction. In addition, the outcomes of this research found that sufferers in the internal fixation 1 had a lower complication rate. It further illustrates the effectiveness and safety of internal fixation treatment. Additionally, bone metabolism is influenced by the body’s inflammatory microenvironment, and osteoporosis is a key factor in the development of ankle fractures, particularly in high-risk populations prone to brittle fractures.^[26]^

## 5. Conclusions

In summary, open reduction and internal fixation for patients with ankle fractures can significantly shorten the time for fracture healing and functional recovery, reduce the incidence of secondary surgery and nonunion after surgery, promote the recovery of ankle joint function, and prevent complications. The incidence of disease is reduced, it is highly safe, and it is worthy of promotion and application. Although certain results have been achieved, our research still has certain limitations, including small sample size, single source, and short time, which may cause certain errors in the study outcomes. In the future, follow-up duration can be extended, and large-scale, multicenter, prospective studies can be conducted, with a focus on additional prognostic factors such as bone health and bone metabolism to enhance the accuracy of research conclusions.

## Author contributions

**Conceptualization:** Yaheng Wei, Zuoming Yang.

**Data curation:** Yaheng Wei.

**Formal analysis:** Yaheng Wei.

**Investigation:** Yaheng Wei.

**Methodology:** Yaheng Wei.

**Resources:** Zuoming Yang.

**Software:** Zuoming Yang.

**Supervision:** Zuoming Yang.

**Validation:** Zuoming Yang.

**Visualization:** Zuoming Yang.

**Writing – original draft:** Yaheng Wei.

**Writing – review & editing:** Yaheng Wei, Zuoming Yang.
